# Injectable biomaterials for vocal fold augmentation: biomechanical principles, clinical outcomes, and emerging regenerative strategies

**DOI:** 10.3389/fbioe.2026.1851460

**Published:** 2026-10-26

**Authors:** Agnes Szalenko-Tokes, Laszlo Rovo, Aniko Keller-Pinter

**Affiliations:** 1 Department of Biochemistry, Albert Szent‐Gyorgyi Medical School, University of Szeged, Szeged, Hungary; 2 Centre of Excellence for Interdisciplinary Research, Development and Innovation, University of Szeged, Szeged, Hungary; 3 Department of Oto‐ Rhino‐Laryngology and Head and Neck Surgery, University of Szeged, Szeged, Hungary; 4 Department of Internal Medicine, Albert Szent-Gyorgyi Medical School, University of Szeged, Szeged, Hungary

**Keywords:** biomaterials, extracellular matrix, injection laryngoplasty, regenerative medicine, viscoelasticity, vocal fold

## Abstract

Disorders of the vocal folds, including atrophy, scarring, and paralysis, frequently result in glottic insufficiency, impaired phonation, and reduced quality of life. Injection laryngoplasty has become a cornerstone treatment for these conditions by restoring vocal fold volume and improving glottic closure through the injection of biomaterials into the thyroarytenoid muscle or paraglottic space. A variety of injectable biomaterials are currently available, including hyaluronic acid, calcium hydroxylapatite, human collagen, autologous fat or fascia, hematogenous biomaterials, and synthetic polymers such as polydimethylsiloxane. These materials differ considerably in their viscoelastic properties, biocompatibility, degradation kinetics, durability, and capacity for host tissue integration. Hyaluronic acid-based fillers closely mimic the mechanical properties of the vocal fold lamina propria and may support extracellular matrix remodeling, whereas autologous fat and fascia offer longer-lasting augmentation with variable long-term persistence. Despite significant clinical improvements in voice quality and glottis competence, their therapeutic effects remain largely structural, providing passive medialization without restoring native tissue microarchitecture or biomechanical function of the vocal folds. Consequently, next-generation biomaterials are increasingly being designed to combine mechanical support with regenerative functionality by promoting extracellular matrix remodeling, preserving mucosal wave propagation, and enhancing long-term tissue integration. This review provides a translational overview of injectable biomaterials for vocal fold augmentation, integrating their clinical performance with biomechanical properties, biological compatibility, and emerging regenerative strategies for functional vocal fold restoration.

## 1 Introduction: injection laryngoplasty for glottic insufficiency

Disorders of the vocal folds, including scarring, atrophy, and paralysis causing glottal insufficiency, remain challenging to treat because of the highly specialized microstructure of the the vocal fold lamina propria and its limited capacity for self-repair (Rosen et al., 2009a). Age-related vocal fold atrophy (presbylaryngis) and post-denervation atrophy result in reduced vocal fold bulk and altered connective tissue properties, resulting in impaired phonation and diminished quality of life (Mallick et al., 2019; Kwon et al., 2010; Watanabe et al., 2023; Spector et al., 2001; Fang et al., 2008). Clinically, affected patients commonly present with breathy dysphonia, reduced vocal intensity, vocal fatigue, and decreased pitch control, and swallowing difficulties rather than respiratory compromise (Mallick et al., 2019). The selection of the treatment depends on the underlying etiology, vocal fold position and mobility, prognosis for neural recovery, time since injury, and patient-specific factors, including comorbidities and treatment expectations.

Injection laryngoplasty has become one of the principal treatments for glottic insufficiency by restoring vocal fold position and volume through minimally invasive augmentation (Figure 1A). The procedure involves the transoral or percutaneous injection of a biocompatible material into the paraglottic space or the thyroarytenoid muscle (Sulica et al., 2010; Ryu et al., 2020; Rovo et al., 2021).

**FIGURE 1:**
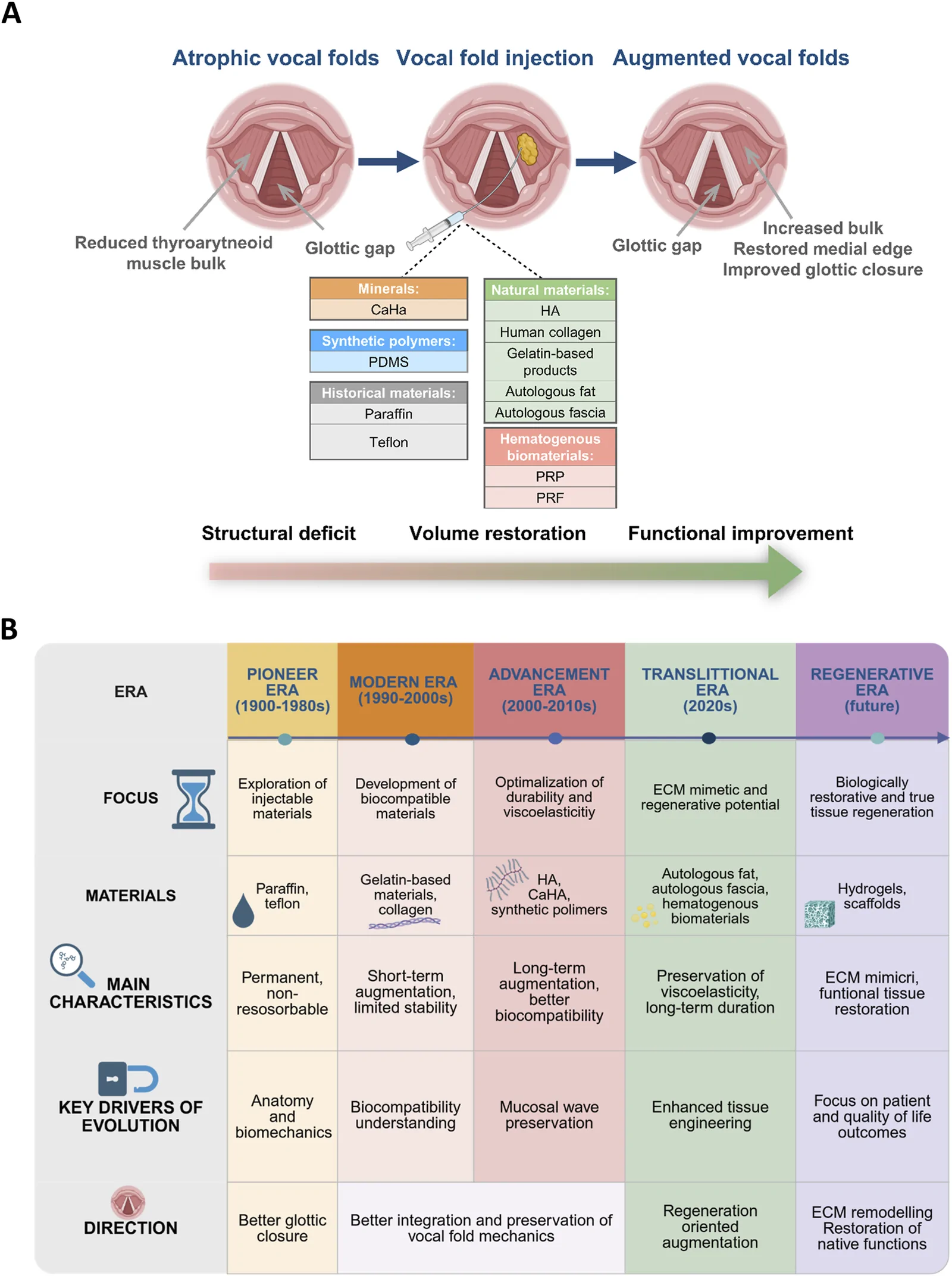
Vocal fold augmentation using injectable materials and the evolution of injectable biomaterials for vocal fold augmentation. **(A)** Schematic representation of injection laryngoplasty for glottic insufficiency. Left, an atrophic vocal fold with reduced thyroarytenoid muscle bulk resulting in incomplete glottic closure. Middle, commonly used injectable biomaterials are delivered into the paraglottic space to restore vocal fold volume and achieve medialization. Right, augmentation increases tissue bulk, restores the medial contour of the vocal fold, and improves glottic closure during phonation. CaHA: calcium hydroxylapatite, HA: hyaluronic acid, PDMS: polydimethylsiloxane, PRF: platelet-rich fibrin, PRP: platelet-rich plasma. **(B)** Evolution of injectable biomaterials used for vocal fold augmentation, highlighting the transition from passive structural fillers toward viscoelastic, ECM-mimetic, and regenerative biomaterials designed to improve tissue integration and preserve phonatory biomechanics. CaHA: calcium hydroxylapatite, ECM:extracellular matrix HA: hyaluronic acid.

Together with medialization thyroplasty, or arytenoid adduction lateropexy, injection laryngoplasty improves glottic closure and voice quality by compensating for tissue loss or paralysis (Ryu et al., 2020; Rovo et al., 2021; Lee et al., 2020; Mortensen et al., 2009). Owing to its safety, technical simplicity, and minimally invasive nature, it has become a widely adopted treatment for mild to moderate glottic insufficiency (Yeou and Shin, 2025). Advances in imaging, delivery techniques, and biomaterials have further expanded its clinical applications (Mallur and Rosen, 2010; Damrose and Berke, 2003). Despite these advances, currently available injectable materials primarily provide structural augmentation and do not reverse the underlying degeneration of the vocal fold extracellular matrix (ECM) or muscle.

Consequently, increasing attention has focused on biomaterials that not only restore vocal fold volume but also promote biological repair and long-term tissue integration. This review provides a translational overview of injectable biomaterials for vocal fold augmentation, emphasizing the relationship between material composition, biomaterial properties, host tissue response, and clinical performance, while highlighting emerging regenerative strategies that may bridge the gap between structural augmentation and functional tissue restoration.

## 2 Biomaterial design requirements for functional vocal fold restoration

The ideal injectable biomaterials for vocal fold augmentation must satisfy a unique combination of biomechanical, biological, and clinical requirements to ensure successful functional restoration of the vocal folds beyond simple volume restoration. Unlike conventional soft-tissue fillers, vocal fold injectables must preserve the highly specialized vibratory behaviour of the superficial lamina propria while simultaneously restoring glottic competence.

A central requirement is rheological compatibility with native vocal fold tissue to support functional integration. The vocal fold is a highly specialized anisotropic and viscoelastic tissue whose layered ECM architecture enables efficient oscillation during phonation (Yeou and Shin, 2025). The human vocal fold lamina propria exhibits highly compliant viscoelastic behaviour with an exceptionally low Young’s modulus, and any injected material must precisely replicate this native elasticity to preserve the delicate mucosal wave necessary for normal voice production (Alipour and Vigmostad, 2012). Injectable materials that are excessively stiff may impair vibration and deteriorate voice quality. Therefore, successful biomaterials should closely reproduce the elasticity and viscosity of the native ECM, preserving the physiological balance required for normal phonatory function.

Furthermore, the optimal clinical injectability and procedural handling are also critical design considerations. Materials should display low shear viscosity to permit injection through fine-gauge needles. Modern hydrogel systems increasingly utilize tunable crosslinking strategies that facilitate minimally invasive delivery while improving *in situ* retention and mechanical durability (Chen et al., 2017).

Biocompatibility represents another essential criteria. In addition to mechanical compatibility, successful vocal fold restoration depends on appropriate host immune modulation and regenerative capacity. Emerging evidence suggests that biomaterials capable of promoting anti-inflammatory, pro-regenerative cellular responses are more likely to support functional healing rather than fibrotic scar formation (Wan-Chiew et al., 2021). Injectable materials should not induce chronic inflammation, fibrosis, granuloma formation, migration, or adverse foreign-body reactions. Because excessive scar formation alters tissue stiffness and disrupts mucosal wave propagation, biomaterials must support favourable host integration while minimizing fibrotic remodelling (Yeou and Shin, 2025).

The biodegradation profile further plays a major role in long-term functional outcomes. Materials that degrade too rapidly may fail to maintain glottic competence, whereas excessively persistent implants may increase tissue rigidity and impair vibration. The biomaterial’s biodegradation rate must be strictly synchronized with the host’s rate of new extracellular matrix (ECM) synthesis to prevent premature volume loss, ensuring a seamless structural transition during tissue regeneration (Abolhasani et al., 2025). There is no universally optimal resorption time, as different clinical indications may require materials with short-, intermediate-, or long-term persistence. Materials that degrade too rapidly may fail to maintain glottic competence, whereas excessively persistent implants can increase stiffness and impair vibration. Consequently, next-generation biomaterials are increasingly engineered with tunable degradation kinetics that synchronize scaffold resorption with endogenous ECM remodelling and tissue regeneration (Chen et al., 2017; Wan-Chiew et al., 2021). Figure 2A summarizes the key translational design parameters and biological characteristics of clinically available and theoretically applicable (but currently unavailable) injectable biomaterials for vocal fold augmentation.

**FIGURE 2:**
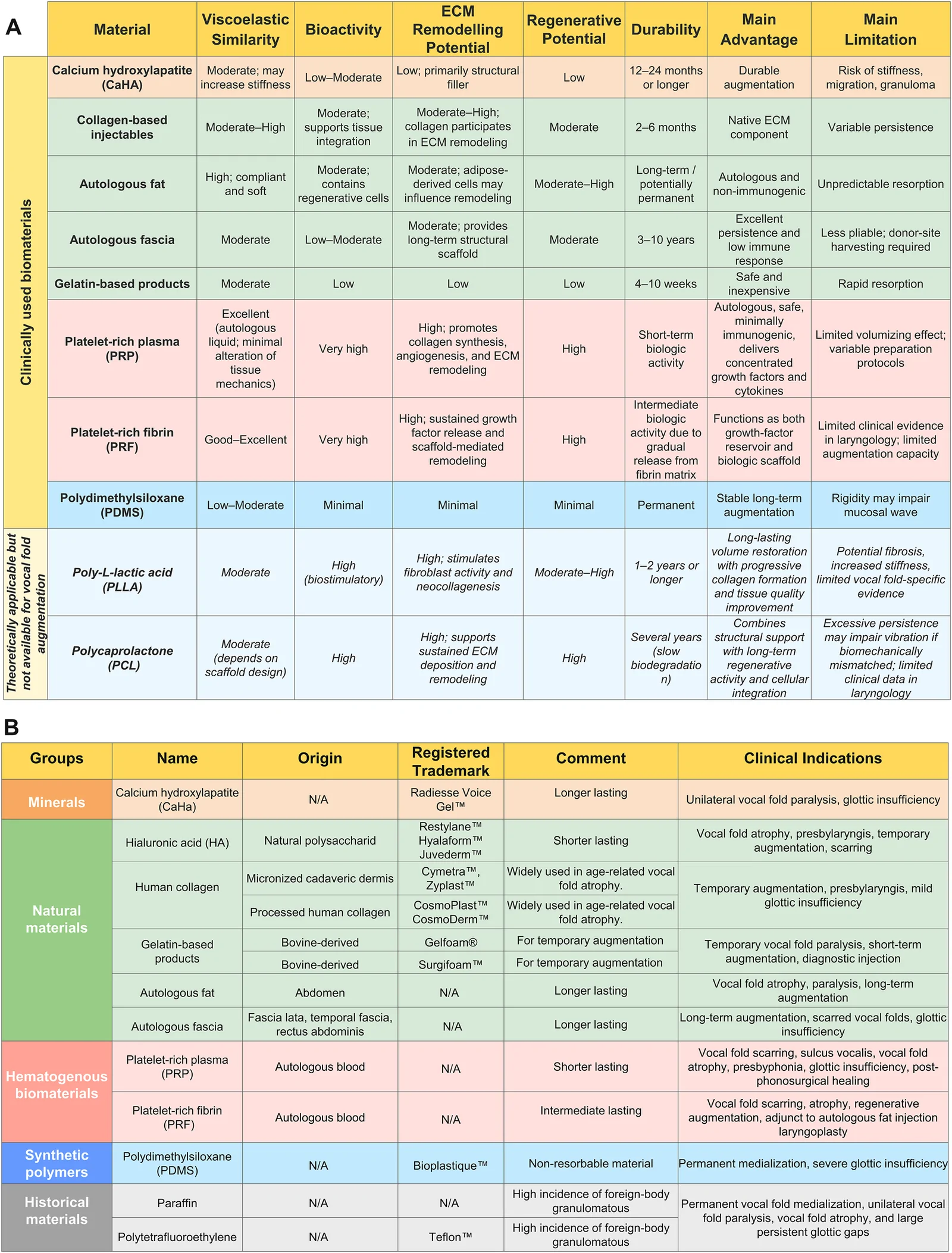
Comparative framework of clinically available and theoretically applicable (not yet clinically available) injectable biomaterials **(A)** and principal clinical characteristics of commercially available injectable materials for vocal fold augmentation **(B)**. ECM: extracellular matrix; N/A: not applicable.

Beyond passive structural augmentation, contemporary biomaterial development increasingly focuses on regenerative functionality. Therefore, future injectable systems are expected to combine mechanical support with biological functionality, facilitating long-term restoration of vocal fold architecture. Current biomaterial design increasingly focuses on recreating the biochemical and structural characteristics of the native ECM. Emerging injectable systems aim to recreate both the biochemical and biomechanical characteristics of the native vocal fold ECM while modulating cellular responses toward pro-regenerative rather than fibrotic healing pathways. Biomaterials incorporating ECM-mimetic architectures, bioactive signalling molecules, extracellular vesicles, or stem cell-based components may therefore provide not only medialization, but also long-term restoration of vocal fold microstructure and function (Yeou and Shin, 2025). The evolution of injectable biomaterials reflects a broader transition from passive structural augmentation toward regenerative and ECM-mimetic strategies aimed at functional tissue restoration (Figure 1B).

## 3 Types of injectable biomaterials

Injectable biomaterials used for vocal fold augmentation can be classified according to their origin as biological, autologous, hematogenous, or synthetic materials. Their clinical performance varies considerably depending on their biomechanical properties, biocompatibility, degradation profile, and persistence within the host tissue. Currently available materials include hyaluronic acid, calcium hydroxylapatite, collagen-based products, gelatin-based materials, autologous fat and fascia, platelet-derived biomaterials, and synthetic polymers (Figures 2A, B). Although these injectables differ in composition and longevity, all are intended to restore glottic competence by improving vocal fold volume and medialization. The following sections summarize the principal characteristics, clinical applications, advantages, and limitations of each biomaterial. Figure 2B summarizes the principal clinical characteristics of commercially available injectable biomaterials used for vocal fold augmentation.

### 3.1 Calcium hydroxylapatite (CaHA)

Calcium hydroxylapatite (CaHA), a naturally occurring mineral, is a long-lasting injectable material that provides durable vocal fold augmentation while maintaining a favorable mucosal vibration profile. It is injected as microspheres suspended in a carboxymethylcellulose-based gel carriers (e.g., Radiesse Voice Gel). Although the carrier gel is resorbed within approximately 1–3 months, the CaHA microspheres continue to provide structural support, resulting in sustained medialization for 12–24 months or longer, with a mean clinical duration of approximately 18 months. Beyond volume restoration, CaHA may promote limited tissue remodeling through interactions with the surrounding ECM However, excessive injection volume or improper placement may increase tissue stiffness and adversely affect vocal fold vibration (Belafsky and Postma, 2004; Carroll and Rosen, 2011; Chhetri et al., 2004; Rosen et al., 2009b; Rosen et al., 2007).

### 3.2 Hyaluronic acid (HA)

Rheological studies have demonstrated that hyaluronic acid (HA)-based injectables most closely replicate the viscoelastic properties of the native human vocal fold. As a natural polysaccharide and a major component of the vocal fold lamina propria, HA not only provides volume restoration but also supports tissue regeneration by supplying structural components for extracellular matrix synthesis. Commercially available HA formulations, including Restylane™, Hyalaform™, and Juvederm™, typically provide clinical benefit for 4–6 months, although some studies report effects lasting up to 12 months. HA fillers are widely favoured for their viscoelasticity, biocompatibility, and reversibility, mimicking the mechanical characteristics of the superficial lamina propria (Molteni et al., 2010; Hertegard et al., 2004; Caton et al., 2007).

### 3.3 Collagen

Human-derived collagen is another biological material for vocal fold augmentation. Available injectable formulations include micronized cadaveric dermis (e.g., Cymetra™), which typically provides augmentation for approximately 2–3 months, and processed human collagen products such as CosmoPlast™ and CosmoDerm™ (Gul et al., 2005). Cymetra is widely used, including in cases of age-related vocal fold atrophy (Remacle and Lawson, 2007; Luu et al., 2007).

### 3.4 Gelatin-based materials

Bovine-derived gelatin-based products, such as Gelfoam® and Surgifoam™, are biocompatible injectable scaffolds widely used for temporary vocal fold augmentation. They are easily administered, provide effective short-term augmentation, and have a favorable safety profile, with effects typically lasting approximately 4–10 weeks (Mallur and Rosen, 2010; Carroll and Rosen, 2010).

### 3.5 Autologous fat

Autologous fat is a widely used injectable material for vocal fold augmentation because of its excellent biocompatibility, safety, and preservation of the viscoelastic properties of the vocal fold (Hsiung et al., 2000; Laccourreye et al., 2003). As an autologous, non-immunogenic material, it provides a biological alternative to synthetic injectables and may produce durable clinical benefits lasting 3 years or longer, leading many authors to consider it a permanent injectable. However, long-term outcomes remain variable because fat resorption is unpredictable. For this reason, overinjection is generally recommended, though this may cause temporary postoperative dysphonia or a foreign-body sensation. Fat is harvested during surgery—most commonly from the periumbilical subcutaneous tissue. In cases of vocal fold atrophy, autologous lipoinjection remains one of the most commonly recommended treatment options (Hsiung et al., 2000; Laccourreye et al., 2003). Autologous fat injection remains an attractive biological alternative, as it is autologous and non-immunogenic; however, its unpredictable resorption and variable long-term outcomes limit widespread adoption.

### 3.6 Autologous fascia

Autologous fascia is an established augmentation material that offers several advantages over fat or collagen, including greater metabolic stability, resistance to resorption, and minimal immunogenicity (Duke et al., 2001; Tsunoda et al., 2005). It can be harvested from several donor sites, including the fascia lata, temporalis fascia, rectus abdominis sheath, or anterior abdominal aponeurosis. Because it is autologous, fascia is inexpensive, readily available, associated with a negligible risk of rejection, allergic reaction, or disease transmission. Clinical studies have reported durable treatment effects lasting approximately 3–10 years (Miura et al., 2024). More recently, combined autologous fat and fascia grafts have been introduced to exploit the complementary properties of both tissues, pairing the regenerative and structural characteristics of fascia with the viscoelastic properties of adipose tissue to improve graft durability and functional outcomes.

### 3.7 Hematogenous biomaterials - Platelet-rich plasma (PRP) and platelet-rich fibrin (PRF)

Autologous hematogenous biomaterials, including platelet-rich plasma (PRP), platelet-rich fibrin (PRF), and related blood-derived products, have emerged as promising regenerative therapies for vocal fold disorders (Bhatt et al., 2023; Woo et al., 2013; Hong et al., 2013). These autologous biomaterials are enriched with platelets and their associated growth factors and cytokines, which stimulate angiogenesis, fibroblast proliferation, ECM remodeling, and tissue repair, thereby supporting the regeneration of injured vocal fold tissue (Everts et al., 2006).

PRP is an autologous platelet concentrate prepared by centrifugation of whole blood to achieve platelet concentrations above physiological levels. The resulting plasma contains a high concentration of growth factors, including platelet-derived growth factor (PDGF), transforming growth factor-β (TGF-β), vascular endothelial growth factor (VEGF), epidermal growth factor (EGF), and insulin-like growth factor (IGF), which promote cell proliferation, angiogenesis, collagen synthesis, and tissue repair. Because PRP is typically prepared as a liquid, it can be injected directly into tissues to stimulate regeneration and healing. In laryngology, PRP has been increasingly investigated for the treatment of vocal fold scarring, sulcus vocalis, vocal fold atrophy, presbyphonia, and glottic insufficiency (Woo, 2023; Santa et al., 2024). Both preclinical and clinical studies have demonstrated that PRP may improve vocal fold pliability, enhance mucosal wave propagation, reduce fibrosis, and promote restoration of the extracellular matrix of the lamina propria, thereby improving voice quality and functional outcomes (Woo, 2023; Santa et al., 2024; Woo et al., 2014).

PRF is considered a second-generation platelet concentrate that differs from PRP in both its preparation and biological properties. It is prepared without anticoagulants, allowing the formation of a dense fibrin matrix that entraps platelets, leukocytes, and growth factors within a three-dimensional scaffold. This fibrin network enables a slower and more sustained release of bioactive molecules while also providing structural support for cell migration and tissue regeneration. Consequently, PRF functions not only as a source of growth factors but also as a biologic scaffold that facilitates wound healing and extracellular matrix remodeling. In vocal fold applications, PRF has been explored as a regenerative biomaterial for scarred and atrophic vocal folds and as an adjunct to autologous fat injection laryngoplasty (Reksodiputro et al., 2021; Dohan et al., 2009; Sudsamai et al., 2024).

### 3.8 Synthetic polymers

Polydimethylsiloxane (PDMS; Bioplastique™) is a non-biodegradable, silicone-based injectable polymer that provides permanent tissue augmentation. Clinical studies have demonstrated favorable long-term safety and durable correction of glottic insufficiency, with stable outcomes reported for follow-up periods of up to 10 months (Bergamini et al., 2010; Sittel et al., 2006; Sittel et al., 2000).

Unlike conventional fillers that primarily restore tissue volume, biodegradable synthetic polymers such as poly-L-lactic acid (PLLA) and polycaprolactone (PCL) possess biostimulatory properties that promote host tissue remodeling and ECM regeneration. Although their application in laryngology remains experimental, evidence from aesthetic and reconstructive medicine has demonstrated favorable safety profiles, sustained tissue augmentation, and improved tissue quality through stimulation of endogenous collagen synthesis (Kwon et al., 2014; Angelo-Khattar, 2022). Since PLLA induces a controlled foreign-body response that stimulates fibroblast activation and collagen deposition (Ponzo et al., 2026); therefore, excessive collagen formation could potentially increase stiffness and compromise mucosal wave propagation, which remains a major concern. The prolonged degradation profile and favorable mechanical properties of PCL make it an attractive scaffold for cell delivery and organized extracellular matrix regeneration (Ponzo et al., 2025). However, its long-term persistence requires careful optimization to avoid excessive rigidity that could impair phonation.

### 3.9 Historical materials

Paraffin and polytetrafluoroethylene (PTFE, Teflon) are now of historical significance only, as their use has been largely abandoned due to the high incidence of foreign-body granulomatous reactions, rigidity and irreversible tissue damage leading to secondary vocal fold volume loss after removal (Mallur and Rosen, 2010).

## 4 Clinical outcomes

Numerous clinical studies have demonstrated that injection laryngoplasty provides significant and measurable improvement in both objective and subjective parameters of vocal function in patients with glottic insufficiency. Acoustic analyses typically reveal reductions in jitter and shimmer, while aerodynamic assessments show improved glottal closure efficiency and decreased mean airflow rate. Patient-reported outcome measures, such as the Voice Handicap Index (VHI), consistently reflect enhanced vocal performance and reduced vocal effort following augmentation procedures (Svejdova et al., 2022; Sielska-Badurek et al., 2017).

Clinical outcomes are strongly influenced by the biomechanical properties, persistence, and tissue integration characteristics of the injected biomaterial. The durability of injectable biomaterials used for vocal fold augmentation varies considerably depending on their composition and intended clinical application. Temporary materials such as gelatin-based agents persist for only weeks providing short-term medialization and are primarily useful in transient paralysis or diagnostic augmentation (Yeou and Shin, 2025). Hyaluronic acid–based injectables are widely favored because their viscoelastic behavior closely resembles that of the native superficial lamina propria, allowing preservation of mucosal wave propagation while providing intermediate-term augmentation lasting 4–12 months (Caton et al., 2007). In contrast, calcium hydroxylapatite, autologous fat, fascia, and synthetic polymers such as PDMS generally provide longer-lasting structural support, although increased durability may be associated with greater tissue stiffness and reduced vibratory pliability (Rosen et al., 2009b; Hsiung et al., 2000; Tsunoda et al., 2005; Sittel et al., 2006).

Despite favorable short-term functional outcomes, injection laryngoplasty remains largely a symptomatic intervention rather than a regenerative therapy. Most currently available biomaterials primarily restore glottic competence through passive medialization without reversing the underlying degeneration of the vocal fold extracellular matrix or muscular atrophy. The injected materials are gradually resorbed, requiring repeat interventions to sustain voice improvement. Consequently, repeat injections are frequently required, particularly when temporary or partially resorbable materials are used. The need for revision or repeat injection depends largely on the injectable material, the underlying pathology, and patient-specific healing characteristics. Repeated procedures are relatively common in patients with persistent unilateral vocal fold paralysis or progressive atrophy. The need for revision procedures depends on the injected material, the underlying pathology, patient-specific healing characteristics, and the progression of tissue degeneration.

Complications are relatively uncommon but may include overinjection, implant migration, inflammatory reactions, granuloma formation, and localized stiffness that can impair mucosal wave propagation (Din-Lovinescu et al., 2021; DeFatta et al., 2012; Enver et al., 2021). These limitations underscore the need for next-generation injectable biomaterials capable not only of providing durable augmentation, but also of supporting biological restoration and long-term preservation of native vocal fold biomechanics.

Although the present review primarily focuses on the physicochemical, biomechanical, and regenerative properties of injectable biomaterials, clinical outcomes may also be influenced by treatment-related factors beyond the material itself. In particular, some patients may receive concomitant substances, combined augmentation agents, repeat injections, or adjunctive procedures, which can affect the interpretation of durability, tissue integration, functional improvement, and adverse events. Therefore, outcomes reported in clinical studies should not always be attributed exclusively to a single injectable material unless the treatment protocol is clearly described. Future clinical investigations should provide detailed reporting of the injected biomaterial, any additional substances or augmentation agents used, injection site and volume, number of procedures, and follow-up duration. Such standardized reporting would improve comparison between studies and support more accurate clinical decision-making.

## 5 Emerging regenerative biomaterials

Recent advances extend beyond conventional fillers toward biomaterials that actively participate in tissue regeneration. Injectable hydrogels incorporating stem cells, extracellular vesicles, growth factors, and biomimetic matrix components are being developed to restore not only glottal closure but also the layered microstructure of the vocal fold. These approaches aim to preserve phonatory biomechanics while promoting regeneration of connective tissue and muscle components. Injectable hydrogels, collagen-derived scaffolds, calcium hydroxyapatite formulations, and decellularized ECM systems are increasingly designed to combine favorable viscoelastic properties with bioactive signaling that promotes organized tissue remodeling, fibroblast activity, and reduced fibrosis (Yeou and Shin, 2025), (Wan-Chiew et al., 2021). Preclinical studies indicate that regenerative biomaterials may achieve more durable tissue integration and improved preservation of vocal fold vibratory properties than conventional injectable fillers, supporting their potential as next-generation therapies for vocal fold insufficiency.

Particular attention has been directed toward hyaluronic acid–based composite hydrogels, decellularized extracellular matrix scaffolds, and bioengineered polymers that provide both structural and biochemical cues. These systems represent a transition from symptomatic augmentation toward functional tissue restoration. Moreover, in the context of vocal fold regeneration, PLLA and PCL may offer future strategies for addressing not only glottic insufficiency but also the underlying degeneration of the lamina propria. However, further preclinical and clinical studies are required to optimize their biomechanical compatibility and ensure preservation of the delicate viscoelastic properties necessary for normal mucosal wave propagation and phonatory function. Therefore, future injectable systems are expected to combine mechanical compliance with biological functionality, serving not merely as passive fillers but as regenerative scaffolds capable of restoring native vocal fold architecture and long-term phonatory function. This regenerative paradigm is increasingly emphasized in contemporary biomaterials research for vocal fold augmentation and tissue engineering.

## 6 The future of injection laryngoplasty

Although dynamic rehabilitation methods - such as selective reinnervation and laryngeal pacing - represent the theoretical ideal for restoring active vocal fold motion in cases of paralysis, their technical complexity and limited clinical applicability suggest that static rehabilitation techniques will continue to dominate the field for the foreseeable future. Advancements in endoscopic imaging and instrumentation are also anticipated to enhance intraoperative visualization and precision of material placement, thereby improving both procedural accuracy and clinical outcomes.

The thyroarytenoid/lateral cricoarytenoid muscle complex and the superficial lamina propria possess highly specific rheological and biomechanical properties that critically influence mucosal wave characteristics, vibratory function, and ultimately, voice quality. Recognizing these factors, future research and development in injection laryngoplasty are expected to focus on biomaterials that not only ensure biocompatibility and safety but also functionally integrate with the host tissue, replicating its biomechanical and viscoelastic behaviour.

Furthermore, the aging global population and the concurrent pressure to reduce healthcare expenditures are likely to drive a continued shift toward minimally invasive, office-based procedures performed under local anaesthesia. These trends align with the broader movement in laryngology toward cost-effective, patient-centred care, where outpatient injection laryngoplasty will remain an essential and evolving component of voice restoration strategies.

## 7 Limitations and the need for biological restoration

While injection laryngoplasty effectively compensates for glottic insufficiency, it does not restore the native biomechanical integrity of the vocal folds (Kimura et al., 2010). Current injectables primarily provide passive medialization without stimulating tissue regeneration. Consequently, there is growing interest in biologically restorative strategies aimed at reversing or mitigating the degenerative processes underlying vocal fold atrophy.

From a clinical perspective, the selection of an injectable biomaterial should be guided not only by its expected duration of effect, but also by the underlying cause of glottic insufficiency, the degree of vocal fold atrophy or scarring, the required precision of placement, and the patient’s likelihood of requiring repeat treatment. Temporary materials may be preferable when neural recovery is expected or when diagnostic augmentation is needed, whereas longer-lasting materials may be considered in stable paralysis or progressive atrophy. However, increased durability must be balanced against the risk of stiffness, impaired mucosal wave propagation, inflammatory reaction, or granuloma formation. In clinical studies, interpretation of outcomes should also consider whether patients received concomitant substances, combined biomaterials, repeat injections, or adjunctive procedures, as these factors may influence durability, tissue integration, and adverse events. Therefore, future clinical evaluation of injectable biomaterials should emphasize standardized reporting of material composition, injection technique, follow-up duration, voice outcomes, complications, and the need for revision procedures.

In summary, injection laryngoplasty remains the principal therapeutic modality for managing vocal fold atrophy, offering a safe, minimally invasive, and effective means of improving phonatory function. Nevertheless, the transient nature of current injectable materials and their inability to induce structural regeneration underscore the ongoing need for biologically based, restorative interventions that can provide durable and physiologically authentic vocal rehabilitation. Recent research has therefore focused on regenerative strategies such as stem cell transplantation, growth factor–enriched matrices, and biomimetic scaffolds that promote extracellular matrix synthesis and myofiber regeneration aimed at restoring the biomechanical and biological integrity of the vocal fold. These strategies aim to achieve true tissue restoration, recreating the layered viscoelastic architecture essential for normal vocal fold vibration.
